# RNA-Seq Based Analysis of Population Structure within the Maize Inbred B73

**DOI:** 10.1371/journal.pone.0157942

**Published:** 2016-06-27

**Authors:** Zhikai Liang, James C. Schnable

**Affiliations:** Center for Plant Science Innovation & Department of Agronomy and Horticulture, University of Nebraska-Lincoln, Lincoln, NE, United States of America; National Institute of Genetics, JAPAN

## Abstract

Recent reports have shown than many identically named genetic lines used in research around the world actually contain large amounts of uncharacterized genetic variation as a result of cross contamination of stocks, unintentional crossing, residual heterozygosity within original stocks, or de novo mutation. 27 public, large scale, RNA-seq datasets from 20 independent research groups around the world were used to assess variation within the maize (Zea mays ssp. mays) inbred B73, a four decade old variety which served as the reference genotype for the original maize genome sequencing project and is widely used in genetic, genomic, and phenotypic research. Several clearly distinct clades were identified among putatively B73 samples. A number of these clades were defined by the presence of clearly defined genomic blocks containing a haplotype which did not match the published B73 reference genome. The overall proportion of the maize genotype where multiple distinct haplotypes were observed across different research groups was approximately 2.3%. In some cases the relationship among B73 samples generated by different research groups recapitulated mentor/mentee relationships within the maize genetics community.

## Introduction

A great deal of biological research depends on reference genotypes that allow researchers around the world on work with material that is genetically identical or nearly identical. For many decades, assessing whether samples labeled as coming from genetically identical sources truly were identical was a costly, time consuming, and often inconclusive process [1] [2]. However, recent advances in genotyping and sequencing technology have revealed a number of cases where sample names and sequence information significantly different stories. One study of human cell cultures found that 18% of cell lines were either contaminated or something entirely different from what they were labeled as [3] with the widely used HeLa cell line being one of the most frequent offenders [4]. Among plants, a recent resequencing study of arabidopsis demonstrated that a line believed to carry a mutation for the ABP1 gene in an otherwise Col-0 background actually contained a wide range of other nonsense and missense mutations as well as a large region on chromosome 3 which came from a different arabidopsis accession [5]. In soybean (Glycine max), segregating variation covering ^~^3.1% of the soybean genome assembly was observed between two sources of the reference genotype used in the construction of the soybean reference genome [6]. Resequencing of multiple plants from a single batch of Columbia-0 seed in arabidopsis identified multiple haplotypes present in areas that summed up to ^~^20% of the total reference genome [7]. The problem of contaminated or mislabeled samples is a very real one in plant biology, and can invalidate the results of experiments in which substantial time and resources have been invested [8].

Here we set out to quantify how severely these issues of divergence among samples labeled as belonging to the same genetic background impact maize (Zea mays), a preeminent model for plant genetics over the past century. Unlike soybean and arabidopsis, maize is a naturally outcrossing species, so reference genotypes must be maintained by manually controlled self-pollination in each generation. Previous studies using small sets of individually scored markers have identified genetic variation between different sources of the same maize inbred [1]. This study focuses specifically on the maize reference genotype B73, which was developed in Iowa and first registered in 1972 [9], widely used in commercial hybrid seed production across the United States for much of the 1970s and 1980s [10] and is represented in the parentage of many elite lines even today [11]. B73 has also been widely used by plant biologists conducting basic genetic research in maize, and was employed in the sequencing and assembly of the first maize reference genome [12].

## Materials and Methods

### Data sources

A search of NCBI’s sequence read archive identified 25 Illumina RNA-seq data sets deposited by 19 independent research group in three countries (Table 1). Two additional RNA-seq data sets were constructed from B73 seed requested from Iowa State and the USDA’s Germplasm Resources Information Network (Control 1 and Control 2 respectively). For these two samples RNA was extracted from 10-day old B73 seedlings grown at the University of Nebraska-Lincoln (Table 1). In four cases where the total amount of data per run was limited (USA 6, USA 8, USA 9 and USA 17), data from multiple sequencing runs labeled as coming from the same sample were grouped together for analysis. In one case, SRR514100, the total quantity of data was excessive, so only 1/10th of the total data set was employed.

**Table 1:** B73 RNA-seq data sets sources.

Sample Name	Run Accession	Library Layout (bp)	Institute
Control 1	SRR3372478	Paired (101)	University of Nebraska—Lincoln
Control 2	SRR3371876	Single (51)	University of Nebraska—Lincoln
USA 1 [13]	SRR651051	Paired (51)	University of Minnesota
USA 2 [14]	SRR1819621	Paired (52)	University of Minnesota
USA 3 [15]	SRR404150	Single (76)	University of Wisconsin—Madison
USA 4 [16]	SRR514100	Paired (151)	University of Wisconsin—Madison
USA 5 [17]	SRR940300	Single (101)	University of Wisconsin—Madison
USA 6 [18]	*SRR*395191, *SRR*395192 *SRR*395194, *SRR*395208	Single (40)	Iowa State University
USA 7	SRR445245	Paired (102)	Iowa State University
USA 8 [19]	*SRR*039505, *SRR*039506	Single (35)	Danold Danforth Center
USA 9 [20]	*SRR*755252, *SRR*762349 *SRR*762350, *SRR*762351 *SRR*764626, *SRR*764627	Single (35)	Danold Danforth Center
USA 10 [21]	SRR1656746	Single (101)	University of Nebraska—Lincoln
USA 11 [22]	SRR1567899	Paired (50)	Iowa State University
USA 12* [23]	SRR504480	Single (100)	University of California—Berkeley
USA 13 [24]	SRR1587038	Single (101)	University of Wisconsin—Madison
USA 14 [25]	SRR1231518	Single (100)	Cornell University
USA 15 [26]	SRR1272115	Paired (50)	DuPont Pioneer
USA 16 [27]	SRR640263	Single (35)	Yale University
USA 17 [28]	*SRR*520998, *SRR*520999	Paired (51)	Cold Spring Harbor Laboratory
USA 18 [29]	SRR536834	Single (76)	Virginia Tech
USA 19 [30]	SRR999052	Paired (50)	Cold Spring Harbor Laboratory
USA 20 [31]	SRR248565	Paired (81)	Stanford University
CHN 1 [32]	SRR491307	Paired (76)	China Agricultural University
CHN 2 [33]	SRR1522119	Paired (102)	China Agricultural University
CHN 3 [34]	SRR910231	Paired (91)	China Academy of Agricultural Sciences
DEU 1 [35]	SRR924107	Single (96)	MPIPZ
DEU 2 [36]	SRR1030995	Single (85)	University of Bonn

* USA 12 harbors a long introgression on chromosome 2.

### Alignment and initial SNP calling

Low quality sequences were removed using Trimmomatic-0.33 with settings LEADING:3, TRAILING:3, SLIDINGWINDOW:4:15, MINLEN:36 [37]. Trimmed reads were aligned to the repeat masked version of the maize reference genome (version B73 RefGen v3) [12] downloaded from Ensemble (ftp://ftp.ensemblgenomes.org/pub/plants/release-22/fasta/zeamays/dna/) using GSNAP in version 2014-12-29 (with parameters -N 1,-n 2,-Q) [38]. Output files were converted from SAM to BAM format, sorted, and indexed using SAMtools [39]. SNPs were called in parallel along ten chromosomes of the maize version 3 using SAMtools mpileup (-I -F 0.01) and bcftools call (-mv -Vindels -Ob).

### SNP list generation

The view function of Bcftools was combined with in-house Python scripts to extract the content of bcf files and classify SNPs based on the number of reference and non-references alleles on every screened SNP locus. In detail, if the total number of reads covering a particular SNP in a particular sample was below 5, then the site was treated as missing data. When 99% reads on the locus of a sample were from the non-reference allele the sample was coded as homozygous non-reference allele. The same criteria were used for calling a site as homozygous reference allele. When the reads containing reference and non-reference alleles totaled more than 90% of all reads and each allele was represented by more than 20% of aligned reads the site was coded as heterozygous. If two or more alleles were present at >1% of aligned reads but the above criteria were not satisfied, the site was also coded as missing data. To reduce the prevalence of false SNPs resulting from the alignment of reads from multiple paralogous loci to a single position in the reference genome, sites which were scored as heterozygous in more than 20% of all genotyped individuals were discarded. In total, 13,360 SNPs were used in downstream analysis. For each of these SNPs, the impact of the SNP on gene function was estimated using SnpEff v4.1 and SnpEff databases (*AGPv*3.26) [40].

### Population structure analysis

The distribution of the three possible genotypes (homozygous reference allele, homozygous non-referenece allele and heterozygous allele) over each of the ten chromosomes of maize was visualized using matplotlib. PhyML 3.0 [41] was used to construct a phylogenetic tree with 100 bootstrap replicates, and 13,360 SNPs in total of 27 data sets. The maximum parsimony tree was constructed using Phylip-3.696 [42] and the full set of 13,360 SNPs with missing data imputed by LinkImpute [43].

### Expression bias test

Individual FPKM (Frequency per kilobase of exon per million reads) value for each gene in each data set was calculated using Cufflinks v2.2.1 [44]. Expression values were averaged across all China and USA South samples (excluded USA 12 sample that contained a unique introgressed region) separately. Only genes with average FPKM values >= 10 in both groups were retained for testing expression bias. The remaining genes were sorted into two groups: genes located in the 7 chromosome intervals where USA South and China showed different haplotypes and genes outside these intervals. The median gene expression value on behalf of each group was used to be compared.

### Origins of haplotype blocks

The origins of haplotype blocks observed in some B73 accessions but not in the published reference genome were investigated using data from diverse maize lines in the HapMap2 project [45]. In order to make comparisons to these data, alignments and SNP calling were performed a second time as above using B73 RefGen v2. All of samples in China or USA North clade were combined to generate a consensus sets of SNP calls with reduced missing data. In examining region c2r2, sample USA 12 was used individually in addition to the combined China and USA North sequences (S1 Fig). In the analysis of region c5r2 (S1 Fig), USA 10, USA 14 and USA 15 were combined to generate a consensus set of SNP calls for the UC-Berkeley clade. The resulting SNP sets were employed for phylogenetic analysis as described above, with the alteration that the an approximate likelihood ratio test (aLRT) method with SH-like was employed. The resulting trees were visualized using FigTree v1.4.2 (http://tree.bio.ed.ac.uk/software/figtree/).

## Results

### Relationship among accessions labeled as B73

**Fig 1:**
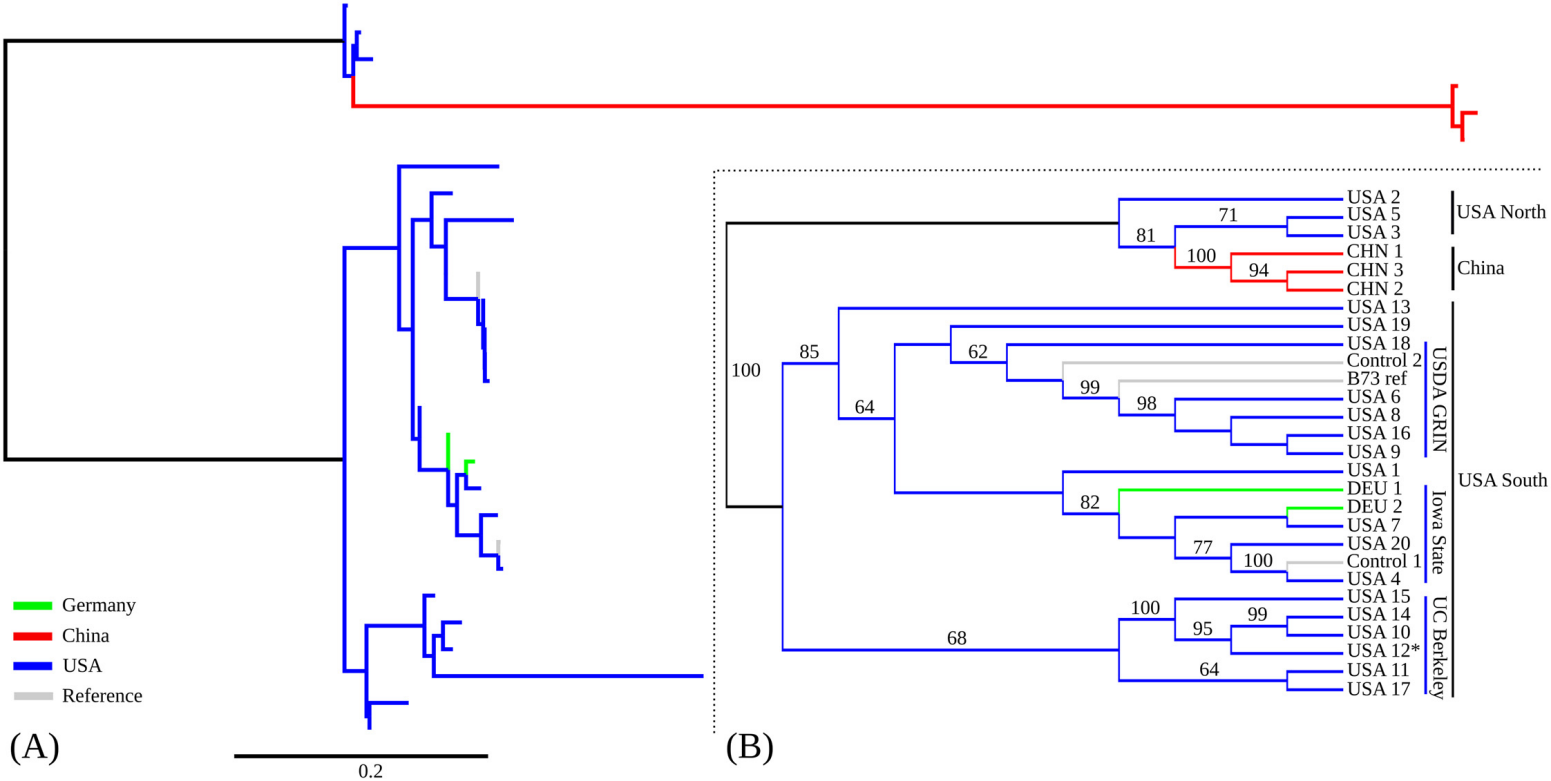
Phylogenetic tree of 27 data sets. (A) Distance-scaled branch lengths; (B) Unscaled tree. Only bootstrap values greater than or equal to 60 are displayed.

After alignment, SNP calling, and filtering (see Methods), a total of 13,360 high confidence segregating SNPs were identified among the 27 RNA-seq samples labeled as B73 employed in this study, substantially lower than the ^~^64,000 high quality SNPs identified by RNA-seq in a population segregating for a single non-B73 haplotype [46]. Phylogenetic analysis identified three distinct clades of samples separated by long branches with 100% bootstrap support (Fig 1; S2 Fig). One clade consisted entirely of Chinese samples, one clade of samples from US research groups from Minnesota and Wisconsin, and the final clade encompassed the majority samples from US research groups as well as all German samples and the published reference genome for B73. We designated these clades “China”, “USA North”, and “USA South” respectively. Notably, the USA North clade is paraphyletic with respect to the China clade, suggesting B73 samples in China are likely derived from this group while both German samples are clearly part of the USA South Clade.

The USA South clade was somewhat arbitrarily divided into three subclades with at least 60% bootstrap support, as well as a number of singleton lineages (USA 1, USA 13, USA 19). Two of these clades contained control samples generated for this study, one from B73 seed requested through the USDA Germplasm Resource Network, and one from B73 seed requested from Iowa State. The subclade containing the known USDA B73 sample also contained the B73 reference genome sequence, consistent with the reported seed source for the B73 used in the construction of the reference genome. The final subclade did not contain any control samples. However, it was notable that four of the six samples placed in this clade originated in research groups whose PIs had conducted either PhD or Postdoctoral training with Michael Freeling at UC-Berkeley, and none of the samples outside of this clade originated in research groups linked to UC-Berkeley. Based on these, we designated the final USA South subclade “UC-Berkeley”. This accessions has also been described as “Freeling B73” [47]. In addition, the three major clades were also recovered in a parallel analysis using a tree generated using maximum parsimony, however the three subclades within USA South subclades were not fully recovered with identical membership (S3B Fig). The consistency index (CI) and retention index (RI) for this tree was 0.825 and 0.861 respectively. Gene flow can product significant amounts of apparent homoplasy when constructing trees from multiple accessions of the same species. Therefore, these values were relatively higher than expected.

### Genomic distribution of within-B73 polymorphisms

The polymorphic SNPs identified in this study could originate from one of several sources including *de novo* mutations or the introgression of non-B73 haplotypes in one or more lineages. SNPs originating from de novo mutations would be expected to show a distribution approximating that of gene density across the maize chromosomes. SNPs resulting from introgression of other haplotypes into B73 should be tightly clustered.

**Fig 2:**
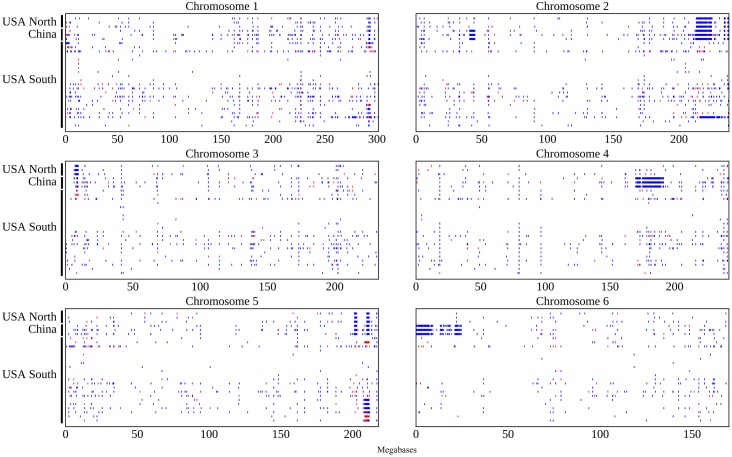
SNP distribution pattern for each of the 27 samples on each of the first 6 chromosomes of maize. Non-reference-like homozygous genotypes are indicated in blue and heterozygous genotypes in red. The sample order from top to bottom on Y-axis in each sub-figure is the same order displayed as in Fig 1B.

When the positions of the SNPs identified in this study were plotted it became clear that 55.3% SNPs identified in this study fall within a small number of dense genomic blocks on chromosomes 2, 4, 5, and 6 (Fig 2). The distribution of non-reference-genome-like haplotype blocks is consistent with the clade relationships identified above. The USA North clade can be defined by a large block of SNPs on chromosome 2, and smaller blocks on chromosomes 2 and 5, all of which are shared with the China B73 clade. In addition to the blocks shared with the USA North B73 clade, samples from the China B73 clade all share a number of additional non-reference-genome-like blocks on chromosomes 2, 4, and 6. There are no non-reference-genome-like blocks shared by all members of the USA South clade, however a single non-reference-genome-like block on chromosome 5 is shared by the UC-Berkeley subclade of USA South. This block appears to share one breakpoint but not both with a block present in the USA North and China samples. Based on the location of this block, it is likely the same divergent haplotype region identified between the B73 reference genome and the B73 sample used to construct HapMap1 [48]. The large block non-reference-genome-like block like SNPs observed only on chromosome 2 on USA 12 can likely be explained by the unique origin of this sample from wild type siblings of knotted1 mutants backcrossed into B73 [49]. The remaining USA South samples, including the USDA GRIN, Iowa State, and German samples do not contain any obvious SNP blocks.

### Functional impact of within-B73 polymorphism

Because the data used here came entirely from RNA-seq studies, our ability to detect SNPs was limited to genes which were consistently expressed at high enough levels to provide coverage of target regions. A total of 25,644 genes were expressed at levels >10 FPKM when at least one of data sets analyzed in this study. Of these genes, 633 (2.5%) fell within regions with non-reference-genome-like SNP blocks in one or more B73 clades. Using SnpEff, we identified 10 cases where SNPs produced “high impact” change such as the gain or loss of a stop code or the alteration of a splice donor or splice acceptor site and 396 cases which produced missense mutations which altered protein sequence. Only three genes with reported mutant phenotypes (whp1, mop1, and gol1) were in these regions, which only constituted at 2.7% of 112 classical identified maize genes with reported mutant phenotypes [50]. However, it must be noted that this is likely an underestimate of the true number of changes, nonsense mediated decay may reduce or eliminate the expression of alleles of genes containing high impact SNPs, reducing the chances these SNPs will be detected from RNA-seq data.

### Impact of within-B73 polymorpism on estimated gene expression

Overall, limited correlation was observed between gene expression level and detected SNP density. The correlation coefficient r between SNP density (number of snps per 1000 bases of exon sequence) and median gene expression across all analyzed datasets was 0.018 and 0.211 for genes outside and inside of block regions respectively (S4 Fig). A previous study found that alignment rate for RNA-seq data from non-B73 genotypes to the B73 reference genome is approximately 13% lower than the alignment rate of RNA-seq data generated from B73 plants [51]. To test whether the introgression of non-reference genome like blocks created a bias towards lower estimated expression of genes in those blocks, for each gene within a block, the the median gene expression value observed across all datasets containing the block was compared to the median gene expression value across datasets where the same genomic region matched the reference genome. The comparison of global patterns across large populations of genes controls for experiment specific changes in the regulation of individual genes. Genes within introgressed regions showed a 5.6% reduction on expression relative to a control set of genes outside introgressed regions in this comparison between B73 USA South and B73 China (see Methods). This reduction was approximately half as large as would be predicted if the reduced alignment rate of data from non-B73 samples resulted solely from the increased difficulty of aligning reads containing SNPs to the reference genome. Potentially, the other half of the reduced alignment rate for non-B73 samples is the result of reads originating from transcripts of lineage specific genes, as previously suggested [51].

### Origins of polymorphic regions in B73 accessions

**Fig 3:**
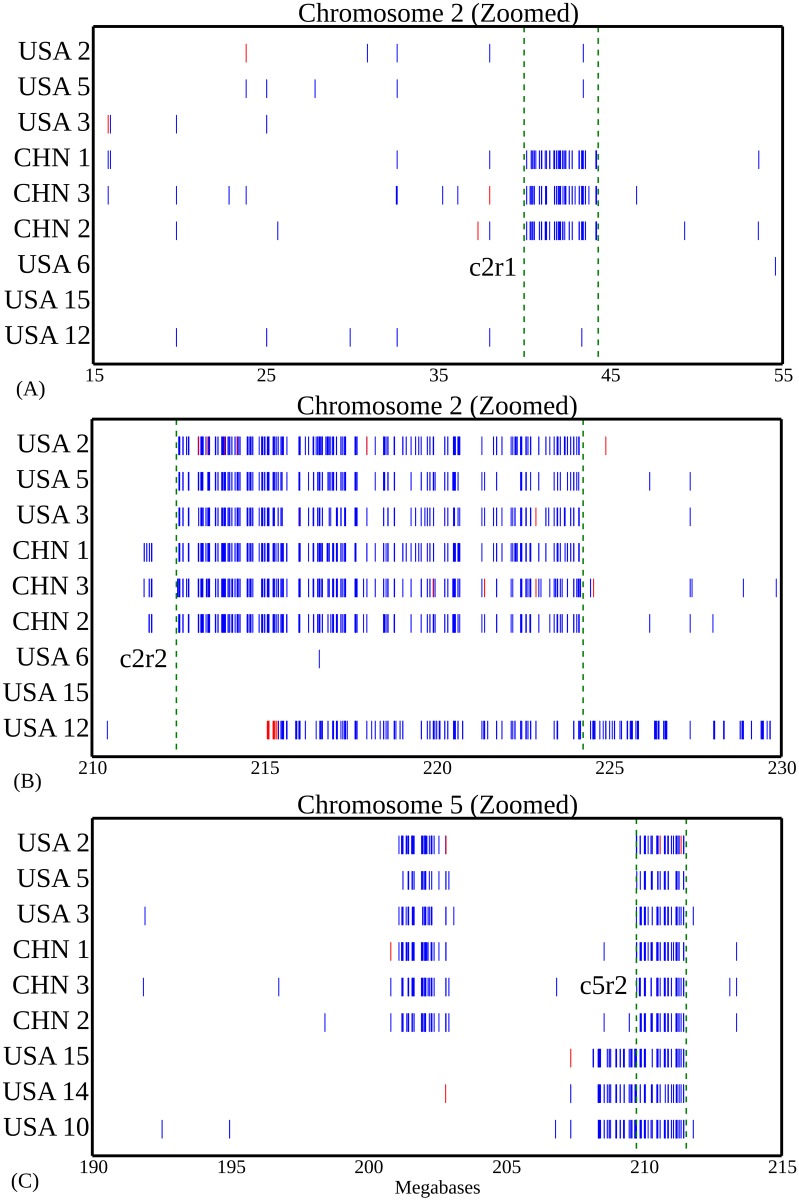
Zoom in on haplotype regions c2r1, c2r2 and c5r2. (A) Haplotype region c2r1 on Chromosome 2; (B) Haplotype region c2r2 on Chromosome 2; (C) Haplotype region c5r2 on Chromosome 5. Non-reference-like homozygous genotypes are indicated in blue and heterozygous genotypes in red. Named haplotype regions are those between the green bars.

**Fig 4:**
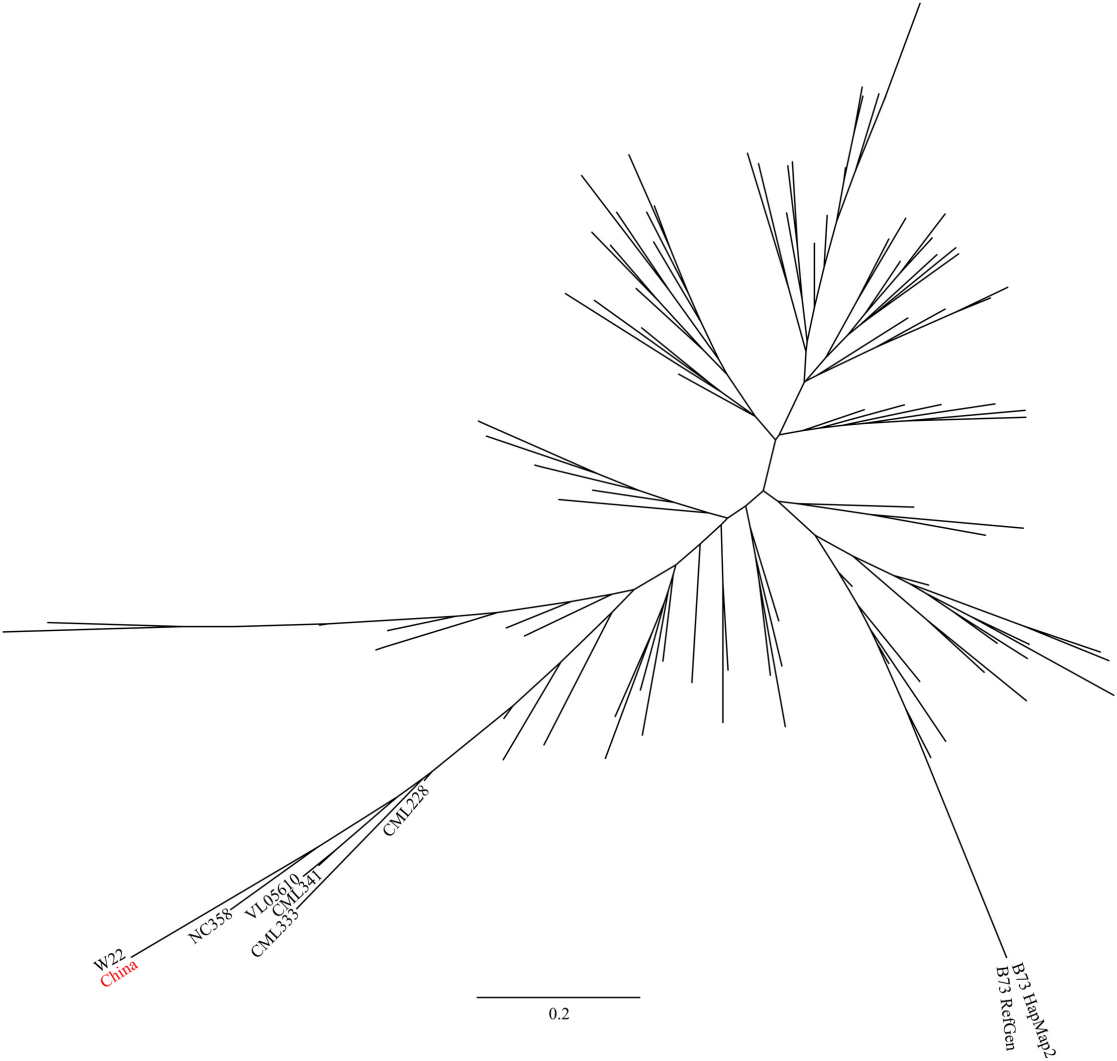
Relationship of the China B73 version of haplotype region c2r1 to the maize HapMap2 varieties.

A total of 7 chromosome intervals (referred to here as c2r1, c2r2, c4r1, c5r1, c5r2, c6r1 and c6r2) containing non-reference genome haplotypes were identified in two or more samples (Table 2; S1 Fig). SNP calls were extracted from individual non-reference-genome-like blocks using the previous version of the maize reference genome (B73 RefGen v2) and compared to genotype calls generated from 103 diverse inbreds resequenced by the Maize HapMap2 project [45]. One example, c2r1 is shown in Fig 3A. The non-reference genome haplotype present in this block for the Chinese samples clusters very closely with W22 (Fig 4), an older inbred developed in Wisconsin which has also been widely used in the maize genetics research community. Analysis of the other six large haplotype blocks produced longer branch lengths relative to the accessions represented in the Maize HapMap2 dataset (Table 2). However, in each case the haplotypes generated from each clade containing a non-reference-genome-like block clustered together, confirming that these regions did not result from parallel introgressions covering the same regions of the genome. Consensus SNP calls from the UC-Berkeley, USA North, and China B73 samples all clustered together with the HapMap2 B73 accession, but not with the B73 reference genome sequence (S8 Fig) which suggests that the source of B73 seed used for HapMap2—like HapMap 1 [48]—likely belonged to the UC-Berkeley subclade. Constraining the c2r2 region to only cover that portion of the genome which contained a block of SNPs in the USA North clade, the China clade and sample USA 12 revealed that USA North and China clustered together while USA 12 was placed at a different location on the tree (S5 Fig). Interestingly, the only separation case of B73 RefGen and B73 HapMap2 in the origin tree of c5r2 indicated B73 seed in HapMap2 came from the UC-Berkeley sub-clade (S8 Fig). In addition, for two cases, c2r1 and c5r2, we validated our haplotype assignments using an orthogonal analytical method, kmeans analysis. SNP data was first imputed using Linkimpute [43], and then grouped into two clusters using kmeans function in R with k = 2 (S1 Table). For c2r1, the analysis was entirely consistent with the results presented above with samples classified as China placed in one cluster with W22 and samples classified as USA North and South placed in the other. For c5r2, as expected all samples classified as China, USA North, and UC-Berkeley subclade were placed in a cluster with the B73 sample resequenced by the Hapmap2 project. In addition, one sample classified as USA South (USA 19) was placed in this cluster. Manual reexamination determined that USA 19 was heterozygous from the c5r2 SNP block (Fig 2).

**Table 2:** Relationship of Non-Reference-Genome Like SNP Blocks to Haplotypes Surveyed by HapMap2.

Genomic blocks	Chr	Start (kb)	Stop (kb)	Closest haplotypes	Branch length	Present in
c2r1	2	40000	44300	W22	0.00000018	China
c2r2	2	212450	224250	BKN010 BKN010 M162W	0.41156403 0.41156407 0.32027864	China USA North USA 12
c4r1	4	169650	191550	CAU178	0.64099035	China
c5r1	5	201200	203000	no single best match no single best match	- -	China USA North
c5r2	5	209732	211540	B73 HapMap2 B73 HapMap2 B73 HapMap2	0.00000001 0.00000021 0.00000001	China USA North UC Berkeley
c6r1	6	120	8800	CML511	0.59542615	China
c6r2	6	20900	24670	OH7B	0.08905230	China

## Discussion

The maize community has long speculated that significant differences exist among B73 from different sources. Several previous studies have confirmed that genetic variation exists between different sources of the same maize inbreds [1] [48] [52], yet due to constraints of cost and seed avaliability these comparisons were genrally able to compare only a small subset of potential seed sources for a given inbred. The avaliability of previously published RNA-seq data sets from a large number of independent research groups has made it possible to conduct a broad survey of the diversity among B73 accessions. No cases of samples which were labeled as originated from B73 but were clearly not B73 based on SNP data were identified in this study. Despite a 40+ generation reproductive history distributed across at least three continents, this analysis shows that 97.7% of the gene space of the maize genome is represented by a single consistent haplotype across all B73 accessions represented here. This compares favorably to approximately 20% of the genome showing multiple haplotypes in a single seed batch of the reference genotype of arabidopsis *Columbia-0* [7]. One potential explanation is that maize geneticists, always aware of the significant risk of pollen contamination, have had to be alert for signs of hybrid vigor or unexpected phenotypes when propogating inbred lines [8].

In soybean, the published reference genome was found to consist of a mosaic of sequences observed in two separate sources of the reference variety and likely is not representative of the haplotype present in any individual plant [6]. In maize, a number of samples classified into the USDA GRIN subclade (Fig 1) are largely consistent with the reference genome suggesting that the maize reference genome sequence likely is representative of a specific plant.

The interspersed SNPs distributed over ten chromosomes of maize may result from *de novo* mutations, segregation of heterozygous loci in the original B73 founder accession [6], or false positive SNP calling errors. However, the majority of polymorphisms identified among B73 samples in this study primarily fell into a small number of dense non-reference-genome-like blocks, consistent with introgression of non-B73 germplasm into a B73 background. It is important to note that the B73 reference genome was sequenced relatively recently compared to the total age of the B73 accession. Therefore, it is not possible to infer whether a given non-reference-genome-like block originated from introgression into the line in which the non-reference-genome SNPs are observed or introgression into the B73 lineage which was ultimately employed in the creation of the B73 reference genome. However, in either case the relatively small size of these non-reference genome like blocks suggests multiple generations of backcrossing to the original B73 line, which would not be consistent with an origin as unrecognized pollen contamination.

Instead we propose a model based on the results from Sample USA 12. USA 12 consists of homozygous wild-type plants selected from family segregating for the Knotted1 [23]. Therefore the block on chromosome 2 (^~^1% of the total maize genome) likely represents residual sequence from the knotted1 mutant donor parent line and is consistent with at least 5 generations of backcrossing (expected contribution of the donor parent = ^~^1.56%). Similar accidental fixations of unlinked regions may have occurred during the intentional introgression of other traits into a B73 background, such as disease resistance genes [53].

The monophyletic placement of Chinese B73 datasets suggests that the B73 seed available in China likely originated from a single transfer from the USA, apparently of seed belonging to the USA North clade and is an indicator of current tight controls on seed import/export which limit the ease with which seed change be exchanged between collaborators in China and the United States. Samples from Germany did not consistently form a monophyletic group. The concordance of academic lineages and genomic relationships in the UC Berkeley subclade acts as a notable positive control. More extensive sampling of B73 samples from many labs which employ this genotype in maize genetics research but have not, to date, published RNA-seq datasets may identify further B73 clades and subclades and additional cases where specific genomic variations have dispersed across the country as graduate students and postdocs leave a given lab for faculty positions of their own.

## Conclusions

The existence of genomic variation among samples labeled as belonging to the same accession creates barriers to reproducibility, one of the core requirements of the scientific method [8]. In this study no examples of sample mislabeling were identified, however the possibility of ascertainment bias, with samples mislabeled as B73 being identified prior to publication must be aknowledged. A number of non-reference-genome-like blocks were identified in B73 samples originating from some sources. These blocks were shown to contain missense and nonsense mutations and measurably lower estimated expression values for genes in these regions. The identification of the relationships among different variants of B73 and the genomic locatons of non-reference-genome-like regions will allow these differences to be controlled for future studies. With the rapid rise of sequencing-based assays such as RNA-seq, the strategy employed here may be a good one to apply in any case where one or more reference genotypes are widely employed in research across institutions, countries, and continents.

## Supporting Information

S1 Fig The 7 named haplotype blocks and SNP distribution pattern across all 10 chromosomes of maize for each of the 27 data sets. (TIF)

S2 Fig A version of Fig 1A drawn as an unrooted tree. (TIFF)

S3 Fig (A) The maximum likelihood phylogenetic tree of 27 data sets by imputed SNP set; (B) One most parsimonious phylogenetic tree of 27 data sets by imputed SNP set.
(TIF)

S4 Fig The scatterplot comparing correlation between SNP density and median gene expression for genes inside and outside of identified block regions. (TIFF)

S5 Fig The phylogenetic tree of c2r2 haplotype region compared to corresponding lines in HapmapV2. (TIF)

S6 Fig The phylogenetic tree of c4r1 haplotype region compared to corresponding lines in HapmapV2. (TIF)

S7 Fig The phylogenetic tree of c5r1 haplotype region compared to corresponding lines in HapmapV2. (TIFF)

S8 Fig The phylogenetic tree of c5r2 haplotype region compared to corresponding lines in HapmapV2. (TIF)

S9 Fig The phylogenetic tree of c6r1 haplotype region compared to corresponding lines in HapmapV2. (TIFF)

S10 Fig The phylogenetic tree of c6r2 haplotype region compared to corresponding lines in HapmapV2. (TIF)

S1 Table Sample clusters in c2r1 and c5r2 haplotype regions. (XLSX)
