# Supplementary figures and images for: RNA-Seq Based Analysis of Population Structure within the Maize Inbred B73
Source: PLoS One. 2016 Jun 27;11(6):e0157942. doi: 10.1371/journal.pone.0157942 (PMC4922647; doi:10.1371/journal.pone.0157942)

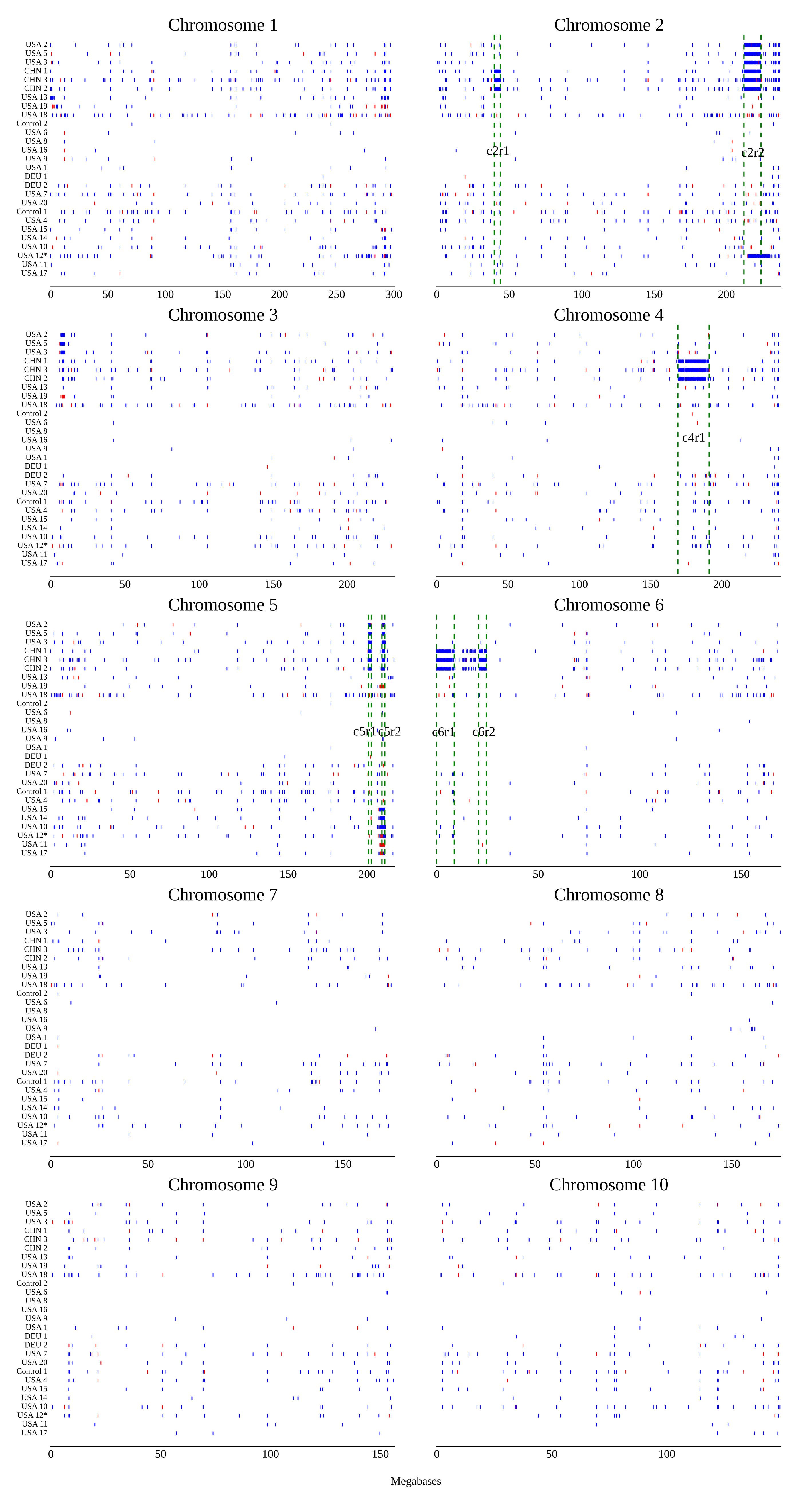

Supplement: S1 Fig — (TIF) [file pone.0157942.s001.tif]

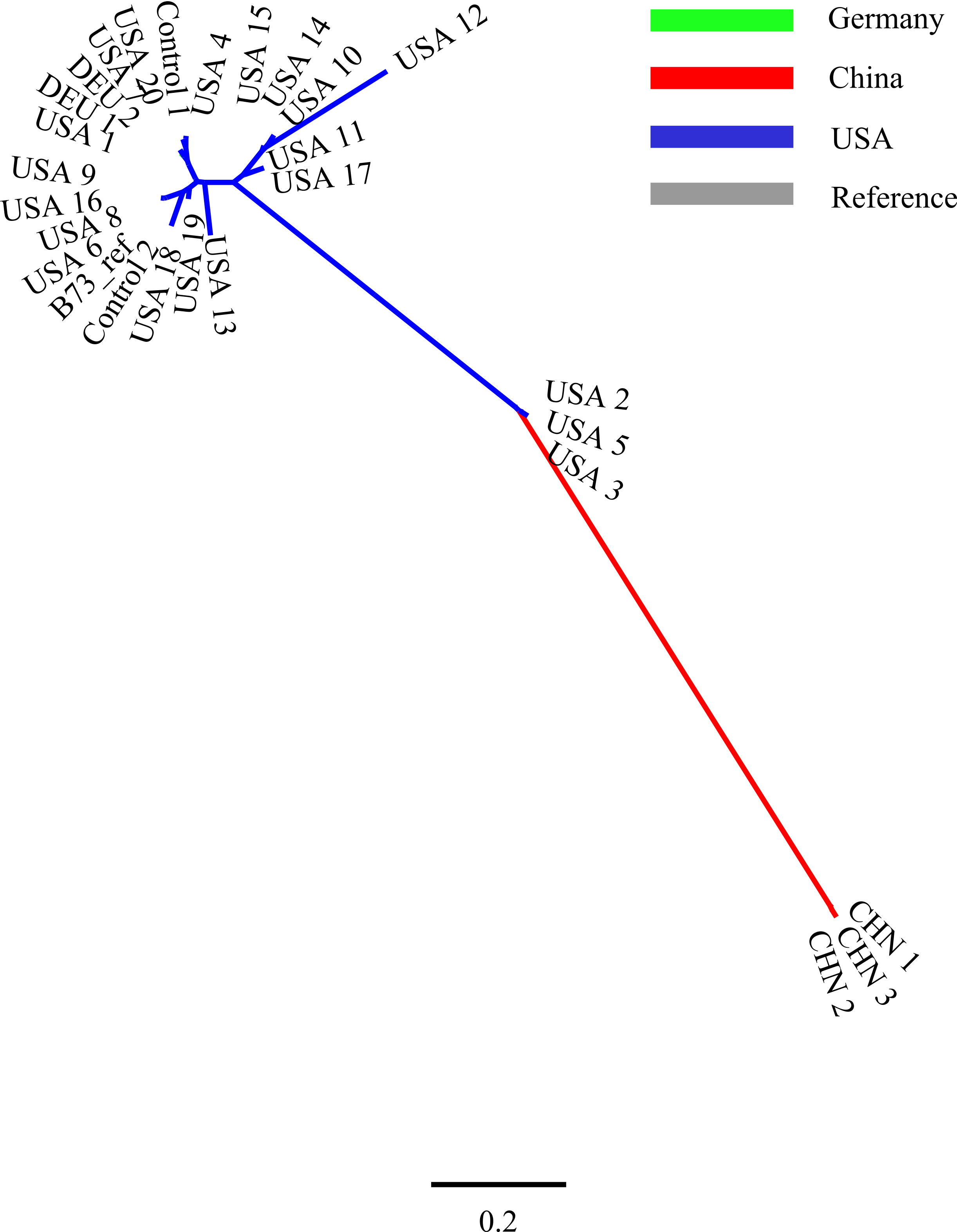

Supplement: S2 Fig — (TIFF) [file pone.0157942.s002.tiff]

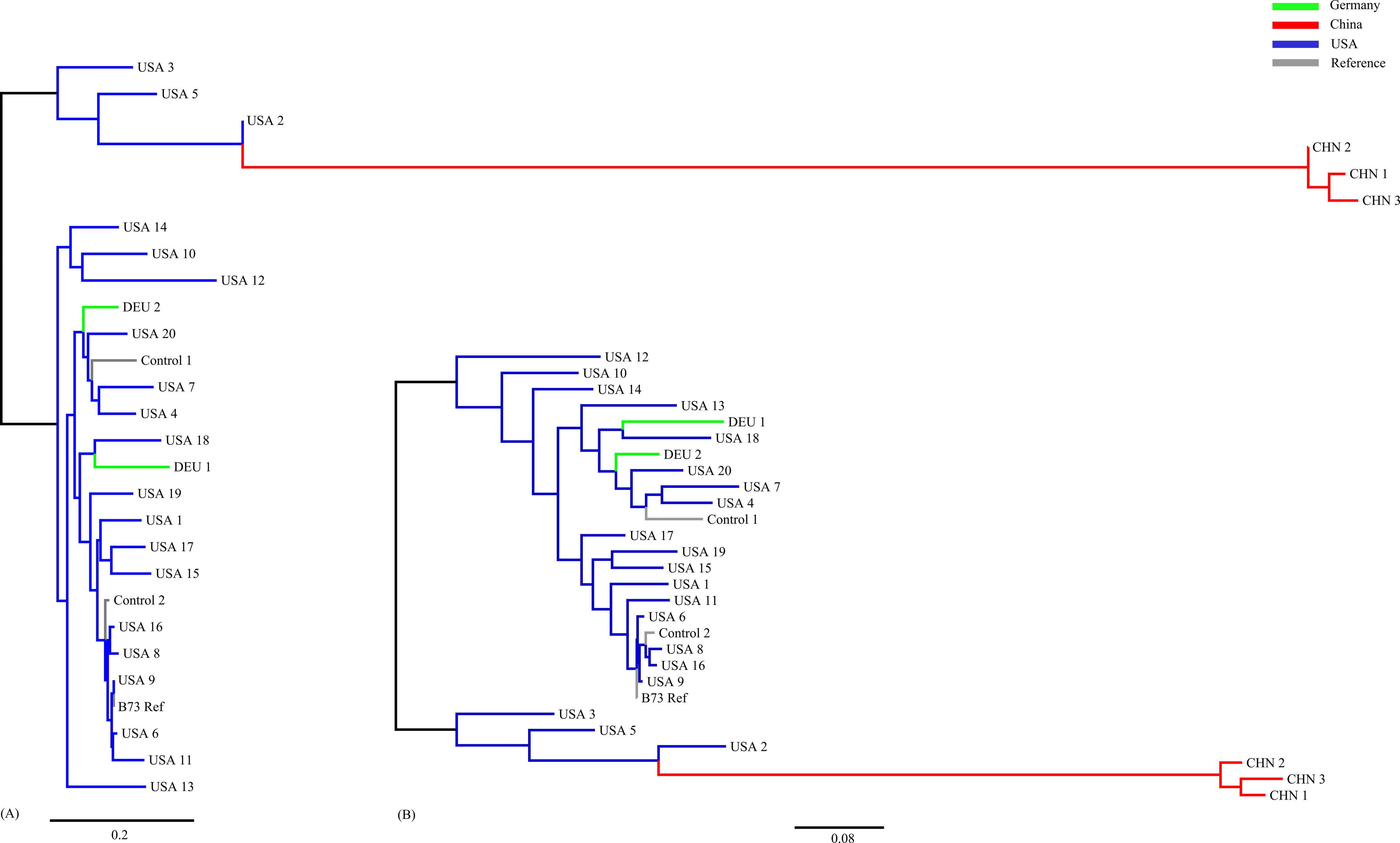

Supplement: S3 Fig — (A) The maximum likelihood phylogenetic tree of 27 data sets by imputed SNP set; (B) One most parsimonious phylogenetic tree of 27 data sets by imputed SNP set. (TIF) [file pone.0157942.s003.tif]

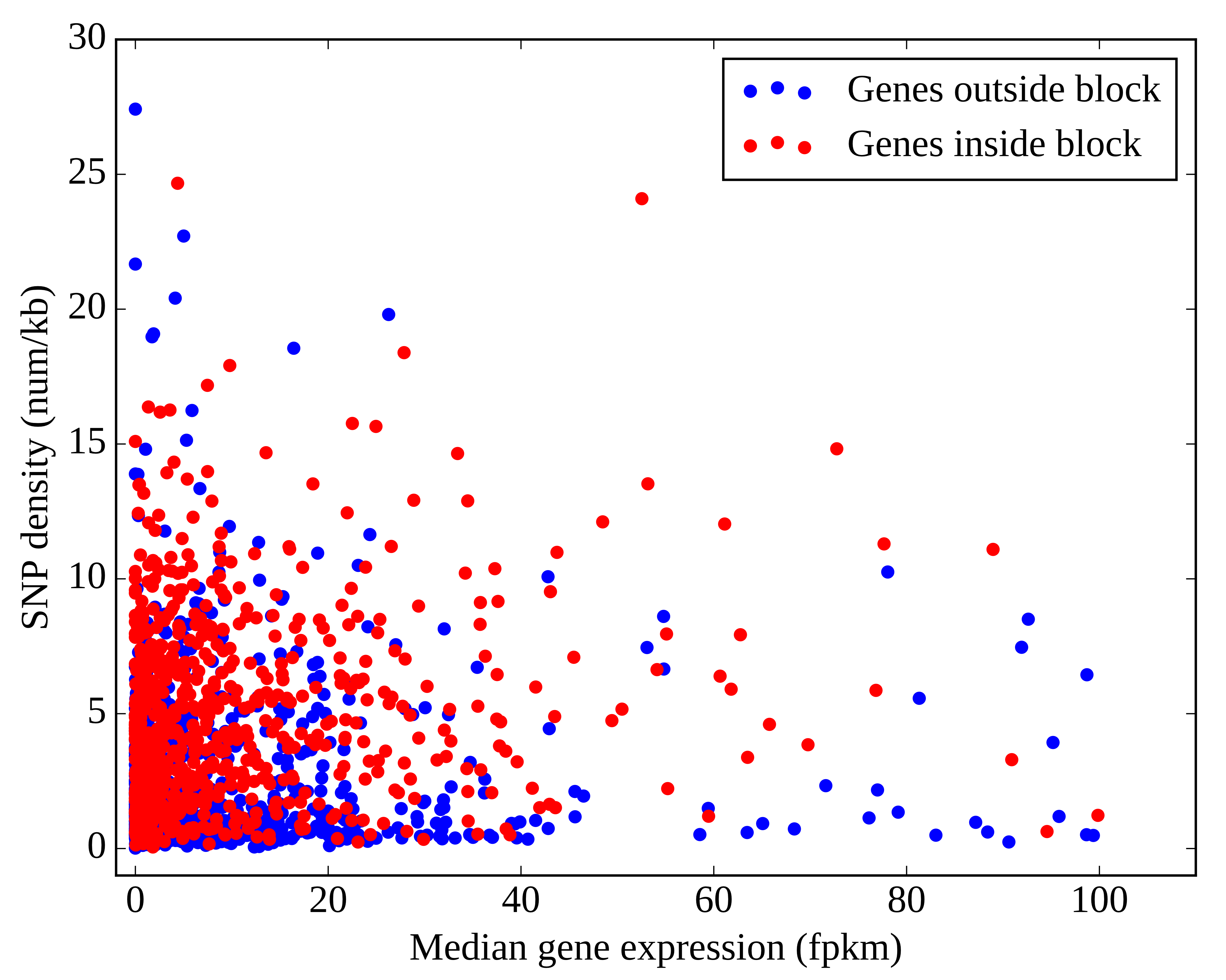

Supplement: S4 Fig — (TIFF) [file pone.0157942.s004.tiff]

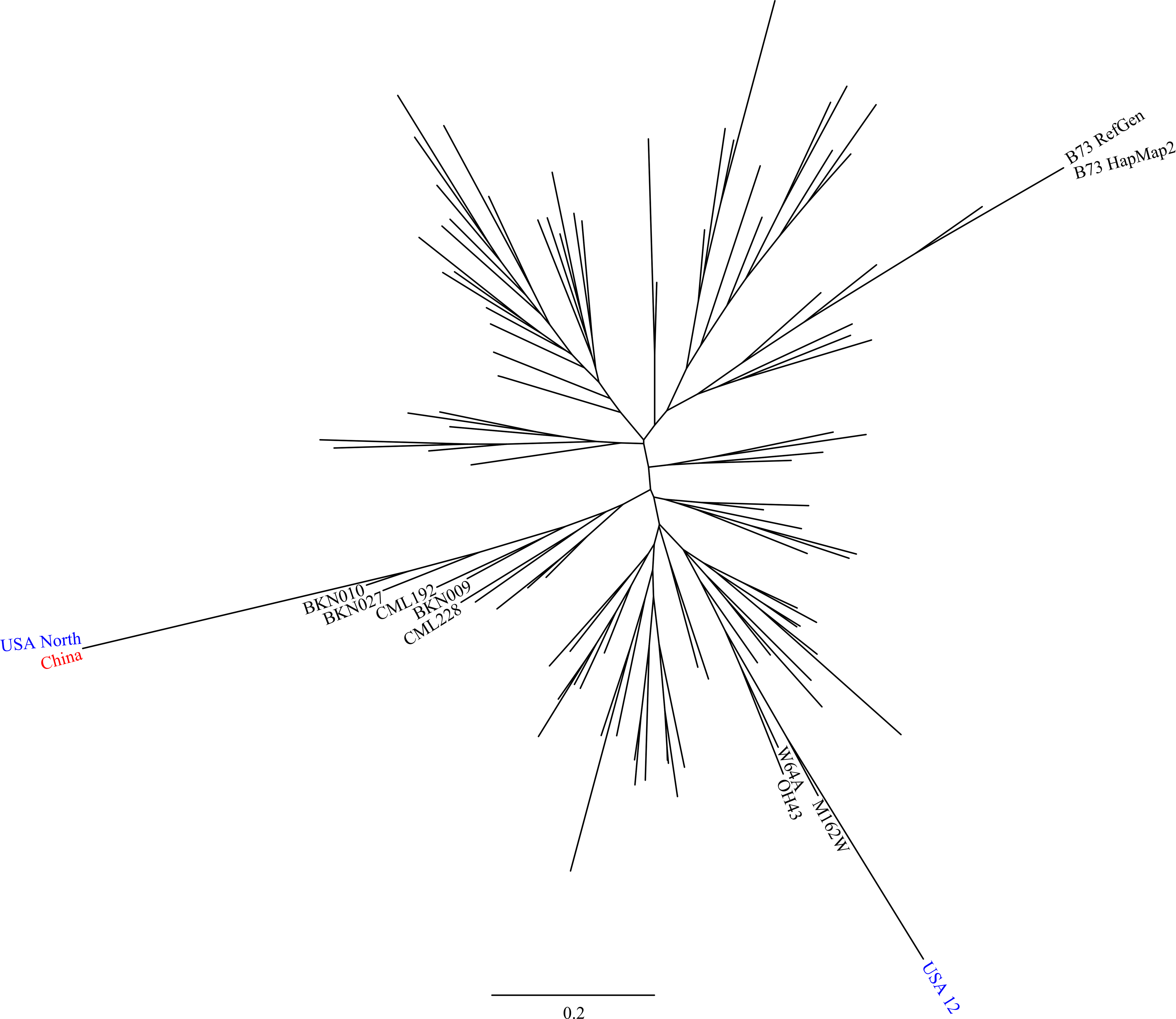

Supplement: S5 Fig — (TIF) [file pone.0157942.s005.tif]

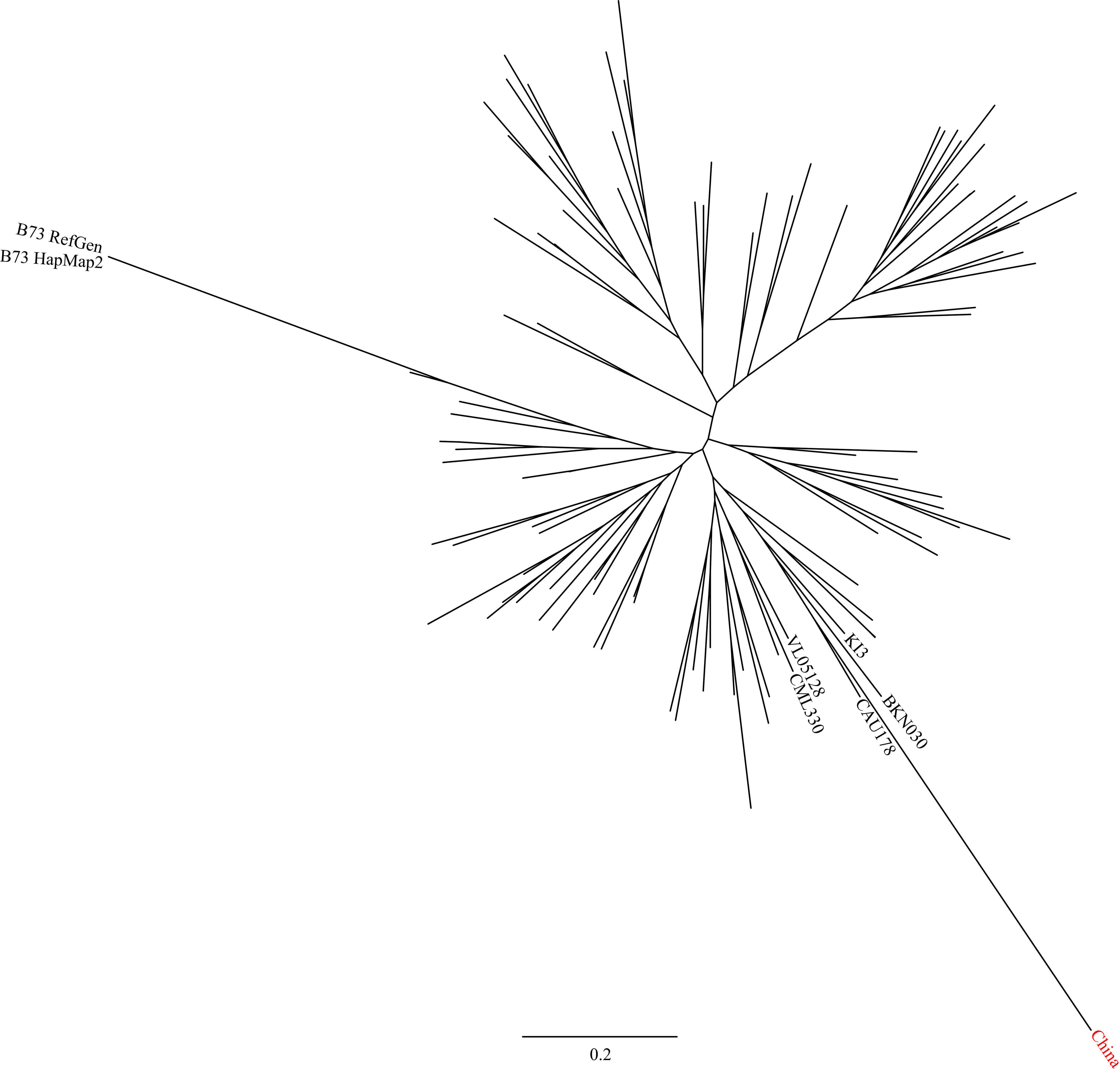

Supplement: S6 Fig — (TIF) [file pone.0157942.s006.tif]

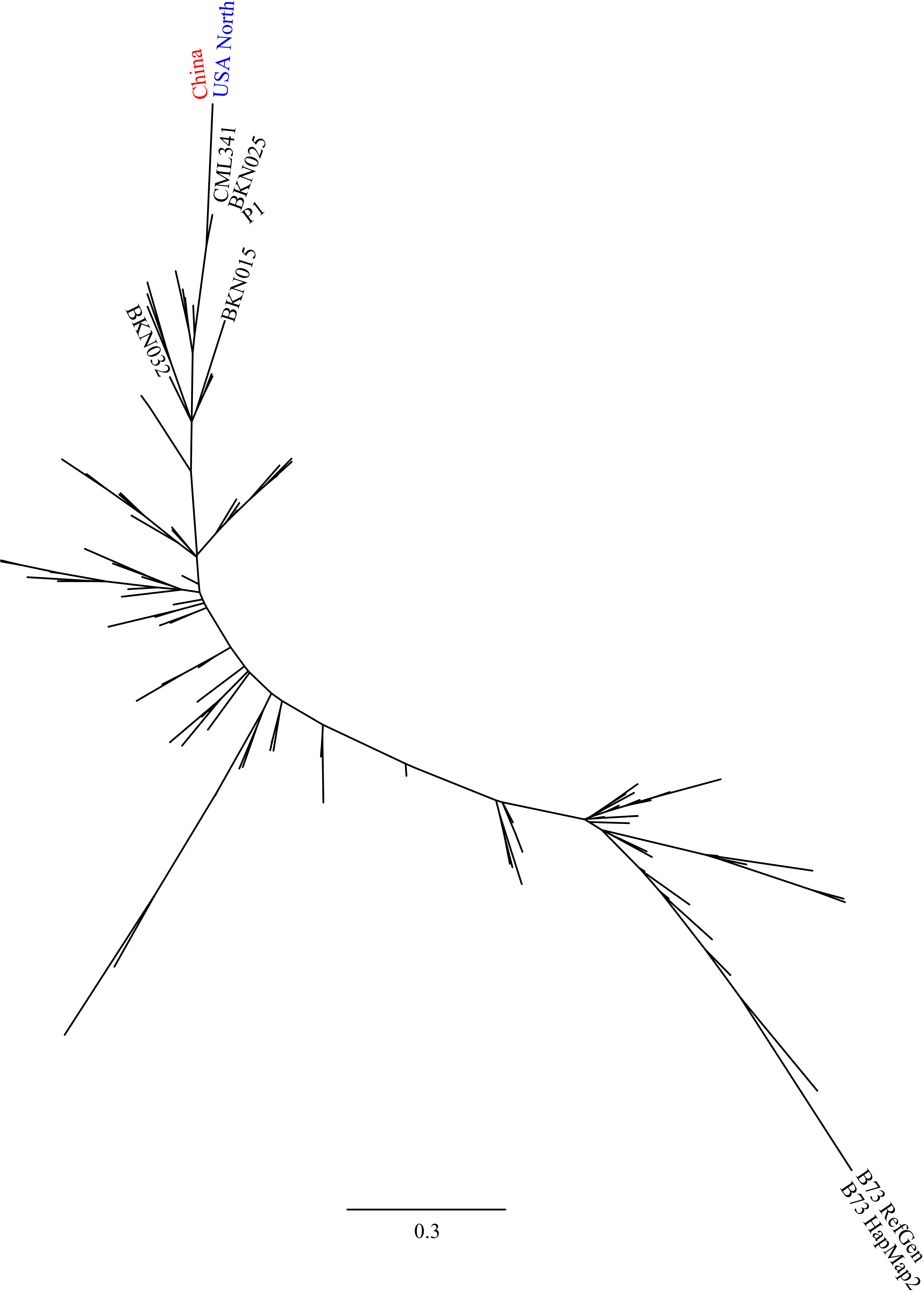

Supplement: S7 Fig — (TIFF) [file pone.0157942.s007.tiff]

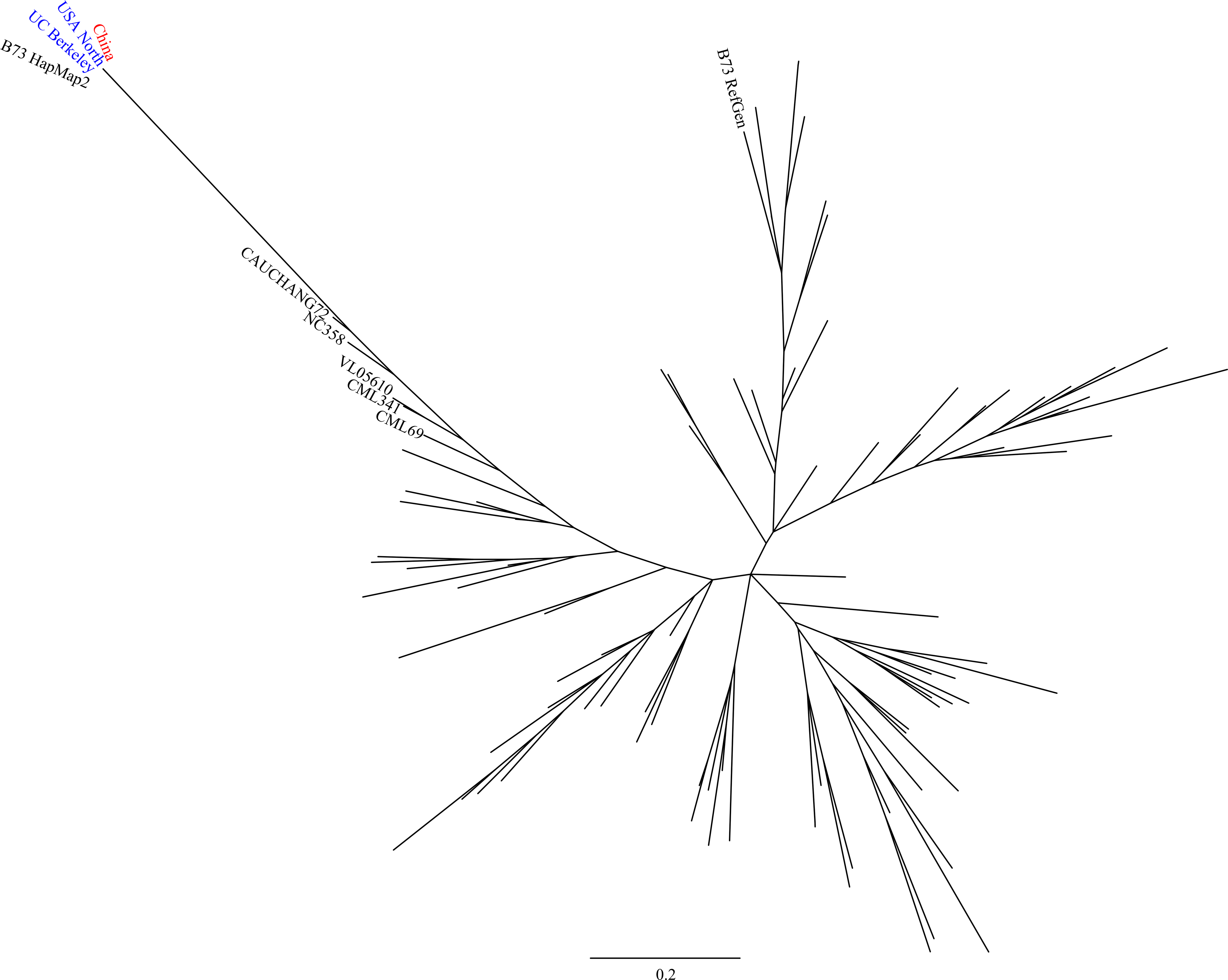

Supplement: S8 Fig — (TIF) [file pone.0157942.s008.tif]

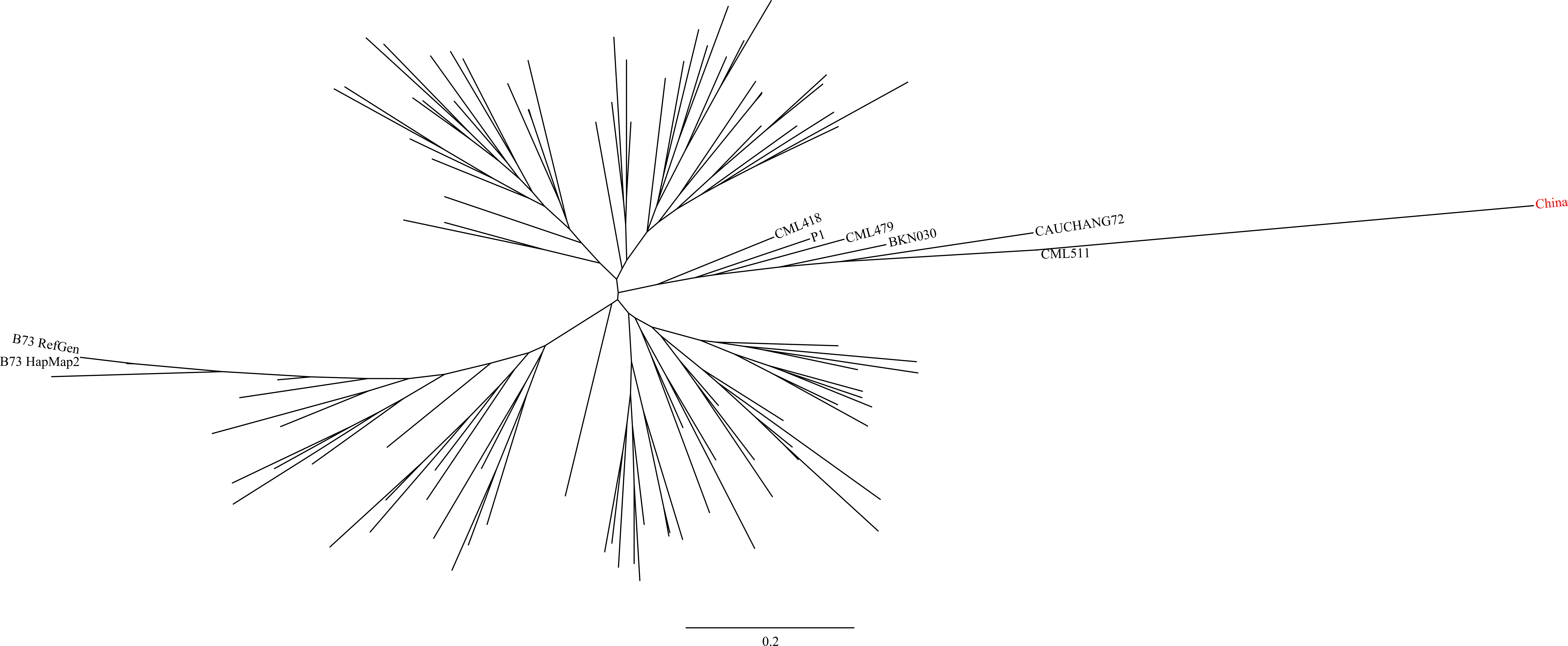

Supplement: S9 Fig — (TIFF) [file pone.0157942.s009.tiff]

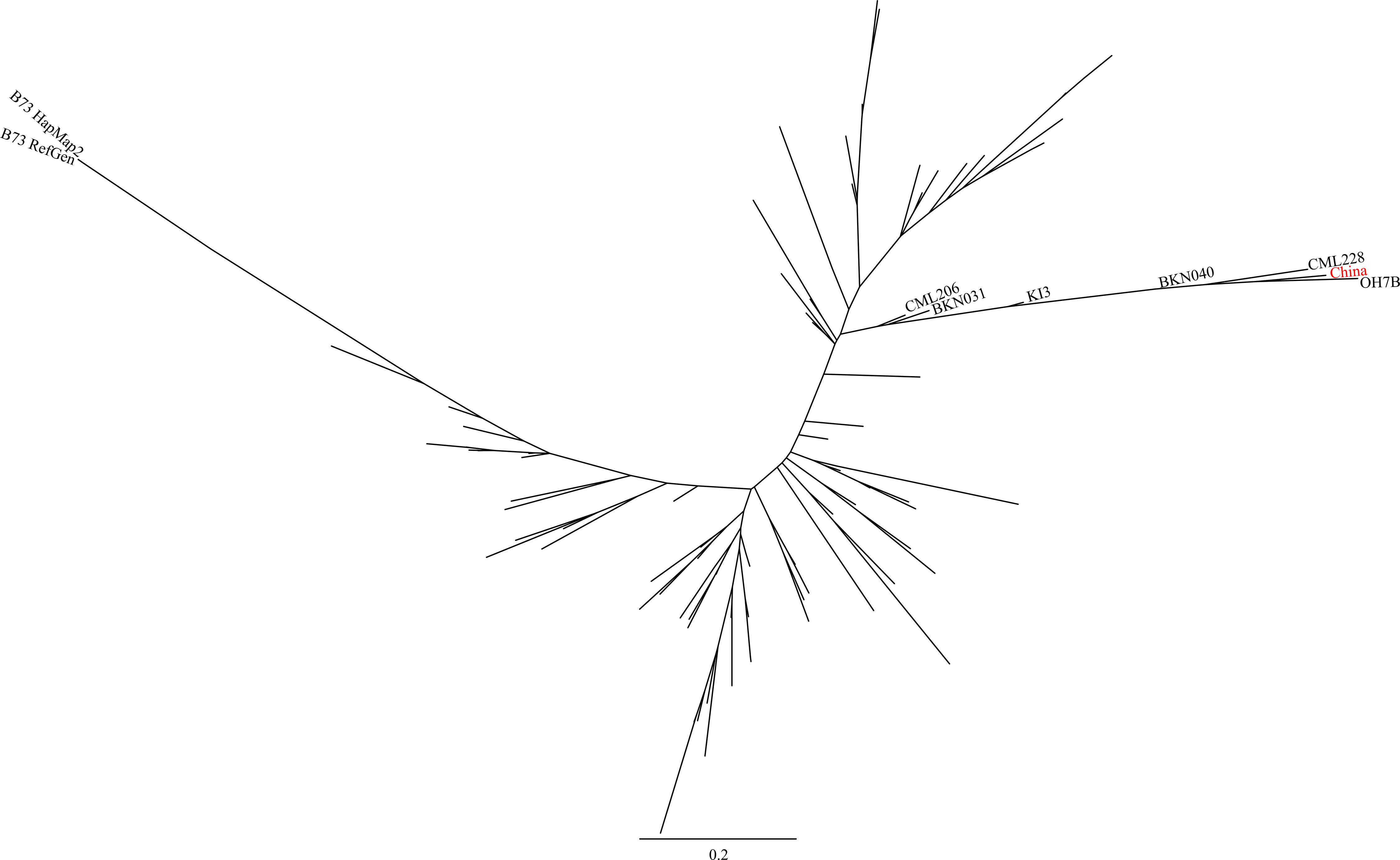

Supplement: S10 Fig — (TIF) [file pone.0157942.s010.tif]
